# Dr. Hillyer’s Address

**Published:** 1859-10

**Authors:** 


### DR. HILLYER'S ADDRESS.

We had hoped to present our readers with a copy of the valedictory by Eben Hillyer, M. D., of Rome, Ga., but in consequence of its late arrival, have been unable to do so in the present No.—a circumstance which we certainly regret, but could not avoid.

